# Machine Learning-Based Multiomics Prediction Model for Radiation Pneumonitis

**DOI:** 10.1155/2023/5328927

**Published:** 2023-02-18

**Authors:** Lu Zhou, Yuefeng Wen, Guoqian Zhang, Linjing Wang, Shuyu Wu, Shuxu Zhang

**Affiliations:** Department of Radiation Oncology, Affiliated Cancer Hospital and Institute of Guangzhou Medical University, Guangzhou, China

## Abstract

**Objective:**

The study aims to establish and validate an effective CT-based radiation pneumonitis (RP) prediction model using the multiomics method of radiomics and EQD2-based dosiomics.

**Materials and Methods:**

The study performed a retrospective analysis on 91 nonsmall cell lung cancer patients who received radiotherapy from 2019 to 2021 in our hospital. The patients with RP grade ≥1 were labeled as 1, and those with RP grade < 1 were labeled as 0. The whole lung excluding clinical target volume (lung-CTV) was used as the region of interest (ROI). The radiomic and dosiomic features were extracted from the lung-CTV area's image and dose distribution. Besides, the equivalent dose of the 2 Gy fractionated radiation (EQD_2_) model was used to convert the physical dose to the isoeffect dose, and then, the EQD2-based dosiomic (eqd-dosiomic) features were extracted from the isoeffect dose distribution. Four machine learning (ML) models, including DVH, radiomics combined with DVH (radio + DVH), radiomics combined with dosiomics (radio + dose), and radiomics combined with eqd-dosiomics (radio + eqdose), were established to construct the prediction model via eleven different classifiers. The fivefold cross-validation was used to complete the classification experiment. The area under the curve (AUC) of the receiver operating characteristics (ROC), accuracy, precision, recall, and F1-score were calculated to assess the performance level of the prediction models.

**Results:**

Compared with the DVH, radio + DVH, and radio + dose model, the value of the training AUC, accuracy, and F1-score of radio + eqdose was higher, and the difference was statistically significant (*p* < 0.05). Besides, the average value of the precision and recall of radio + eqdose was higher, but the difference was not statistically significant (*p* > 0.05).

**Conclusion:**

The performance of using the ML-based multiomics method of radiomics and eqd-dosiomics to predict RP is more efficient and effective.

## 1. Introduction

Radiotherapy is one of the most important treatment methods for lung cancer. However, radiation-induced pulmonary injury is the main limiting factor and the most common complication of thoracic tumor radiotherapy. An accurate prediction model is desired to clarify the risk factors of RP, guide the design of radiotherapy treatment plans, and prevent high-risk patients in advance.

In the clinical, the incidence of RP is correlated with the dose to the lung tissue. The lung volume within which the dose is greater than xGy (Vx) from DVH, such as V30, V20, and V5, is widely used for RP prediction. Afterward, some studies have shown a strong correlation between these image features (such as CT image density or 18F-fluorodeoxyglucose uptake in positron emission tomography) and tissue heterogeneity at the cellular level [1–3]. With the development of radiomics, the digital image processing and ML techniques can be applied in medical image analysis [4–10]. Cunliffe demonstrated the ability of radiomics to provide a quantitative, individualized measurement of patient lung tissue reaction to RT and assess RP development [11]. Based on these studies, the dosiomics method has been proposed, which attempts to extract the spatial features from dose distribution to construct a prediction model [12–16]. Liang et al. applied the framework of radiomics on dose distribution and demonstrated that the dosiomic features improve the prediction ability efficiently [17–19].

Most of the previous studies used the radiomic features of the whole lung, combined with the dose-volume factors and some clinical treatment characteristics, to train and obtain the prediction model of RP. Some studies showed that RP occurs outside the tumor, and lungs that receive low doses (5 or 20 Gy) are associated with RP [20, 21]. Thus, the region of interest was the lung-CTV in this study. The single dose characteristics only utilize partial information on the dose distribution without information regarding the spatial relationship of voxels. The same Vx value may be obtained from voxels that are spread throughout the OAR or connected voxels, so the Vx's biological impact may differ [22–29]. The same absorbed dose with varying fractions of treatment, different time intervals, and different numbers of irradiation per day will represent different biological doses and result in other biological effects. The higher dose of each fraction, the greater the biological effect on tissue, especially for the late reaction tissues such as the lung [30, 31]. In the clinical, the technology of hyper-fractionated or large-fractionated may be adopted for some patients or changed to hyper-fractionated after a certain dose of conventional radiotherapy [30]. Besides, the radiation dose per fraction (Fx) has been shown to be a significant factor in RP [29]. Previous studies only used the dosiomic features from the physical dose distribution. In this study, the equivalent dose of 2 Gy fractionated radiation (EQD_2_) was used to solve the problem of equivalent dose calculation for different fractionated radiotherapy. The physical dose distribution of lung tissue was converted to the EQD_2_ distribution, and the eqd-dosiomic features were extracted from the EQD_2_ distribution using the framework of radiomics. Finally, the predictive performance of four ML models, including DVH, radio + DVH, radio + dose, and radio + eqdose, was compared.

## 2. Methods and Materials

### 2.1. Patient Database

In this study, 350 patients diagnosed with nonsmall cell lung cancer who received radiotherapy in our hospital from 2019 to 2021 were collected retrospectively, in which 91 patients met the selection criteria. At first, all patients were pathologically diagnosed with nonsmall cell lung cancer and had definite TNM stages of lung cancer (Stage I, II, III, IV). Second, each patient underwent two high-resolution diagnostic CT scans without surgical intervention. The first was before radiotherapy, and the second was within six months after radiotherapy. All patients were treated entirely with intensity-modulated radiation therapy (IMRT), and the plans were designed using the pinnacle treatment planning system (V9.10). The slice spacing of the planning CT image was 5 mm, and the grid spacing of dose calculation was 3 × 3 × 3 mm. The classification of RP was carried out according to the RTOG classification standard for acute radioactive lung injury. The endpoint of this study was grade ≥ 1 RP. The clinical and treatment characteristics of patients are shown in Table 1, and the details of 91 patients are available in the supplementary file 1.

### 2.2. Data Acquisition and Dose Conversion

**Figure 1:**
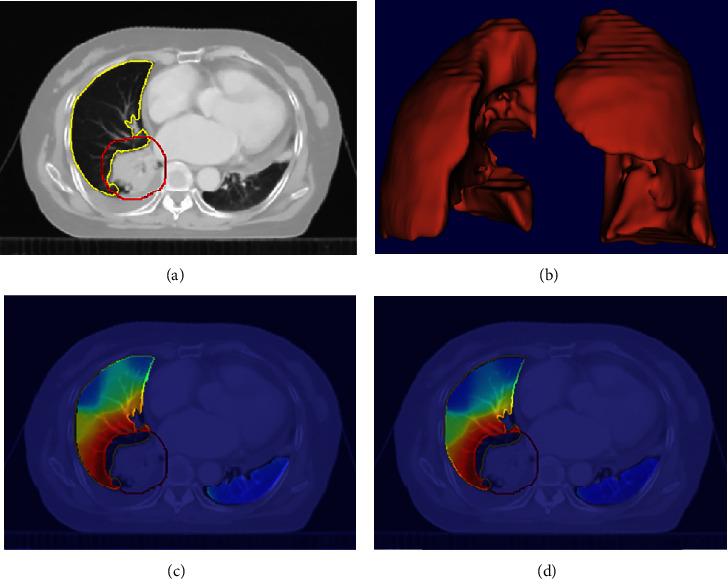
The results of data acquisition and dose conversion: (a) the delineated CTV and lung in CT image, the red is CTV, and the yellow is lung; (b) the 3D volume of lung-CTV; (c) the overlapping of physical dose distribution and CT image; (d) the overlapping of EQD_2_ distribution and CT image.

In the study, the self-developed automatic radiotherapy plan analysis software was used to obtain the 3D volume (in Figures 1(a) and 1(b)) and the original 3D dose distribution (in Figure 1(c)) of the lung-CTV area from the Pinnacle treatment planning system (V9.10).

In the clinical, it is necessary to maintain the total dose required for equivalent biological effects when changing the conventional treatment plan. The EQD_2_ is the dose that causes an equivalent biological effect to conventional 2 Gy fractionation. The problem of equivalent dose calculation for different fractionated radiotherapy was solved using the EQD_2_ model [30–34]. The linear quadratic (L-Q) model is derived directly from the cell survival curve. The concept of the isoeffect dose and the mathematical derivation formula based on the L-Q model can be used to standardize and compare biological doses of radiotherapy with different fractions [35]. The formulation of EQD_2_ was as follows:

(1) $$\begin{array}{c} {EQD}_{2}=D\frac{d+\left(\alpha /\beta \right)}{2+\left(\alpha /\beta \right)}\text{,} \end{array}$$

where D represents the total dose, and *d* is the fractional dose for spatial points in the lung-CTV area. The (*α*/*β*) reflects the radio-biological characteristics of tissue. The value of (*α*/*β*) is larger for early-response tissue and smaller for late-response tissue. As the lung is late response tissue, the value of (*α*/*β*) is set to 4.5 [31]. Then, formulation (1) was used to convert the physical dose into the EQD_2_. The EQD_2_ distribution was shown in Figure 1(d).

### 2.3. DVH Parameters

In the clinical, the lung volume within which the dose is greater than xGy (Vx) is widely used for RP prediction. In this study, the V30, V20, and V5 were extracted as DVH features for all patients.

### 2.4. Feature Preprocessing

At first, the radiomic, dosiomic, and eqd-dosiomic features were extracted from the CT image, the physical dose, and the EQD_2_ distribution of the lung-CTV area. All of the features were extracted by Pyradiomics 3.0.1 [36]. The wavelet filter was used for radiomics calculations. Eight feature groups (a total of 863 radiomics features), including first-order statistics (18 features), shape-based (14 features), gray level co-occurrence matrix (GLCM, 24 features), gray level run length matrix (GLRLM, 16 features), gray level size zone matrix (GLSZM, 16 features), gray level dependence matrix (GLDM, 14 features), neighboring gray-tone difference matrix (NGTDM, five features), and wavelet features (744 features) were extracted, respectively.

Secondly, the data were randomly divided into two sets, with 70% for the training set and 30% for the testing set. The synthetic minority oversampling technique (SMOTE) was used to prevent overfitting by the unbalanced ratio in the training set.

Thirdly, the random forest algorithm was used to select the features. The random forest model performs well because of the sample randomness, feature randomness, and integration strategy. After data preprocessing, the random forest model was performed to calculate the importance of independent variables and filter out the redundant information [37].

At last, the fivefold cross-validation was used to reduce the overfitting in nonlinear regression in the study. The training data were randomly divided into five folder sets, iteratively performed in each model, and the metric of interest was calculated on each validation set. Then, the five values of the metric were averaged to get the training AUC. The testing set was used to independently evaluate the validity of the prediction model by the testing AUC, accuracy, precision, recall, and F1-score.

### 2.5. Prediction Model

This study established four ML models, including DVH, radio + DVH, radio + dose, and radio + eqdose. In addition, eleven classifiers, including logistic regression, ridge, SVM, perceptron, decision tree, random forest, KNeighbors, passive aggressive, GaussianNB, multinomialNB, and Adaboost, were used to establish the multiomics prediction model of RP. The hyper-parameters for the eleven classifiers are available in supplementary file 2. Python 3.7.6 and scikit-learn 0.24.2 were performed for feature extraction, data preprocessing, and ML modeling.

## 3. Result

### 3.1. Feature Importance

**Figure 2:**
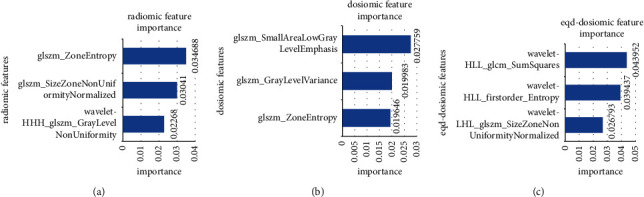
The three most important features: (a) the radiomic features, (b) the dosiomic features, and (c) the eqd-dosiomic features.

Six features were selected from the radiomic, dosiomic, and eqd-dosiomic features, respectively. As shown in Figure 2, most of the selected features were lung texture features, which represent periodic changes and structural rules in the spatial domain of images and can fully reflect the heterogeneity of tissues [38, 39]. The gray level size zone matrix (GLSZM) features quantize the region of continuous pixels in the image, including the features describing the distribution of small or large areas and low or high gray regions. It significantly affects the representation of texture consistency, nonperiodic, or speckled texture and has better performance in image texture analysis [40]. The gray level co-occurrence matrix (GLCM) describes the joint distribution of the grayscale of two pixels with a specific spatial position relationship, which reflects the comprehensive information of the direction, adjacent interval, and variation amplitude of image gray [36].

**Figure 3:**
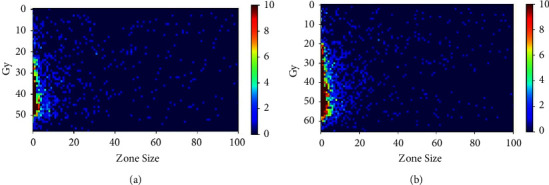
The gray level size zone matrix (GLSZM) of the 3D physical dose distribution (a) and the EQD_2_ distribution (b).

Figure 3 shows the GLSZM derived from the 3D physical dose distribution and EQD_2_ distribution for an example patient. The information on GLSZM for EQD_2_ is more abundant than the physical dose.

### 3.2. Model Performance

**Figure 4:**
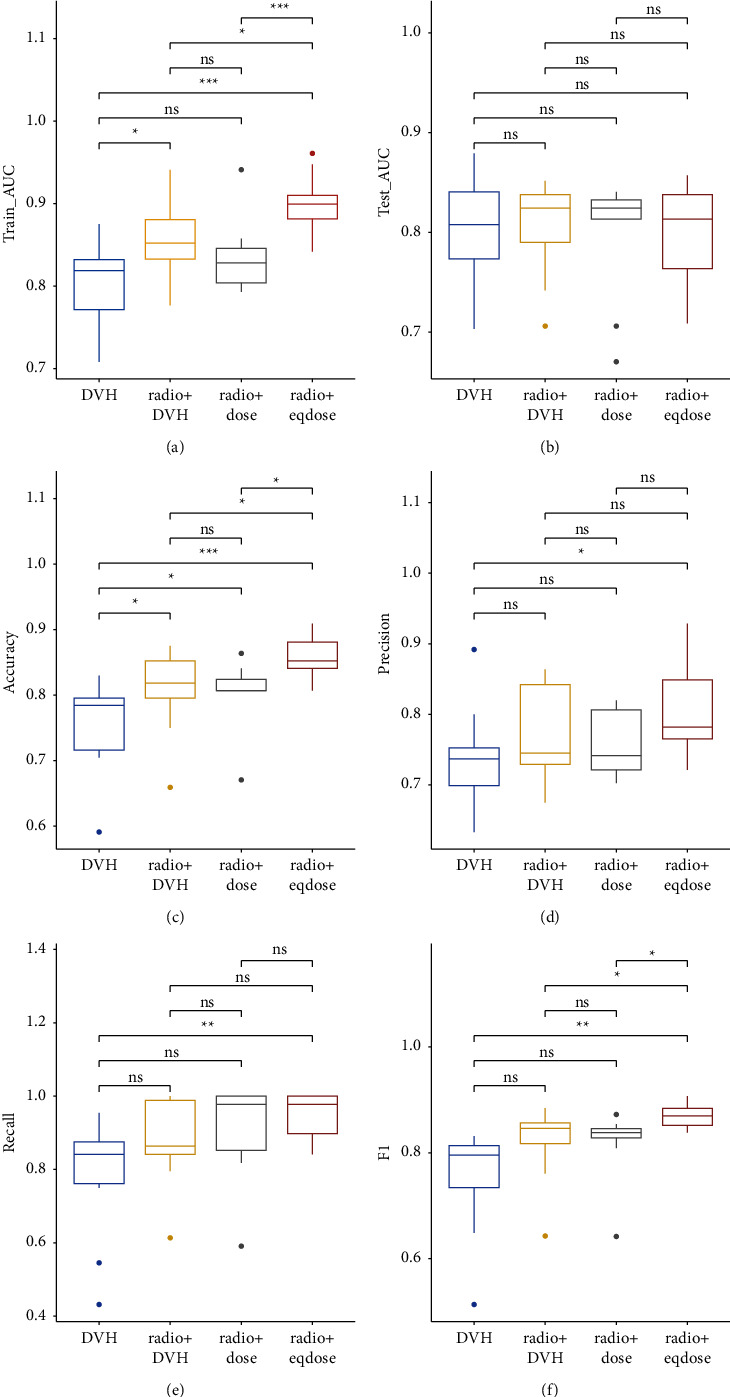
The performances of four ML models under 11 classifiers: (a) is the training AUC, (b) is the testing AUC, (c) is the accuracy, (d) is the precision, (e) is the recall, (f) is the F1-score. In the figure, ^*∗*^indicates *p* < 0.05, ^*∗∗*^indicates *p* < 0.01, ^*∗∗∗*^indicates *p* < 0.005, and ns indicates *p* > 0.05.

The statistical analysis of the performance of four ML models under 11 classifiers is shown in Figure 4 and Table 2. The evaluation indicators of each classifier can be found in supplementary file 3. The statistical analysis was done to prove that the proposed method's performance in this study was not affected by different classifiers. The training AUC is the mean value of five folds for the training set. The testing AUC, accuracy, precision, recall, and F1-score are values for the testing set. The Student's *t*-test was used to calculate *p* values.

Compared with the DVH, radio + DVH, and radio + dose models, the training AUC value of radio + eqdose was higher, and the difference was statistically significant (*p* < 0.05). Besides, for four models, the testing AUC average values were almost 0.8, and the difference was less than 1%. The results showed that the four ML models could predict RP well, among which the radio + eqdose performed better. Compared with the other three models, the accuracy of radio + eqdose was higher, and the difference was statistically significant (*p* < 0.05), representing that the radio + eqdose model could more accurately predict whether patients will suffer from RP. The precision rate reflects the model's ability to distinguish negative samples, the recall rate demonstrates the model's ability to identify positive examples, and the F1-score considers both the classification model's precision and recall. Compared with the other three models, the F1-score of radio + eqdose was higher, and the difference was statistically significant (*p* < 0.05). Therefore, the multiomics method of radiomics and eqd-dosiomics can improve the predictive performance of RP.

## 4. Discussion

In the clinical, the doctors will determine the dose and time segmentation mode of radiotherapy according to the size and lethal dose of the tumor, the tolerated dose of normal tissue, and the normal tissue distribution around the target area. The technology of hyper-fractionated or large-fractionated may be adopted for some patients or changed to hyper-fractionated after a specific dose of conventional radiotherapy [30]. The radiation dose per fraction (Fx) has been shown to be a significant factor in RP. However, previous studies did not consider the effect of fraction dose. In this study, we investigated the effect of different fractional doses on dosiomics. The EQD_2_ model was used to convert the physical dose to the isoeffect dose. The radiomic, dosiomic, and eqd-dosiomic features were extracted, respectively, from the image, the dose distribution, and the isoeffect dose distribution of the lung-CTV area.

In this study, most of the selected features are texture features extracted from the original image characteristics through some calculation and stored in an intermediate matrix. Then, a series of statistics are defined on this intermediate matrix as the texture features. In the GLCM, matrix elements ij represent the times a voxel with gray level *i* is a neighbor of voxels with gray level *j* [36]. The GLCM_Sum Squares is a measure of the distribution of neighboring dose pairs about the mean dose, which reflects the information regarding the spatial relationship of voxels. The GLSZM features describe the regional consistency of the dose distribution textures, in which pixel ij is the number of times a 3D zone with dose *i* has a size *j* [40, 41]. The feature GLSZM_Size Zone nonuniformity normalized measures the variability of size zone volumes throughout the EQD_2_ distribution, with a lower value indicating more homogeneity among zone size volumes. The first-order entropy specifies the uncertainty in the dose distribution. The GLSZM_Zone entropy measures the uncertainty and randomness in the zone sizes and dose levels distribution. Lower zone entropy might indicate a more uniform dose in the region of interest. The GLSZM_Gray level variance calculates the variance in dose values for the zones [36]. Therefore, we speculated that the occurrence of RP is related to the local dose variation in lung tissue. Besides, the GLSZM_Small area low gray level emphasis measures the proportion of the joint distribution of smaller regions with lower dose values in the physical dose distribution. This feature indicates that the low-dose region of lung tissue is associated with the development of RP.

Bin Liang also showed that the higher the local dose variation within the ipsilateral lung and the greater the low-dose region of total lungs, the greater probability of RP incidence [17–19]. The DVH parameters, such as V5, V20, and V30, are just accumulated doses of a specific volume of lung tissue that cannot reflect the local dose variation. However, the texture features show information regarding the spatial relationship of dose distribution for radiotherapy response prediction. As shown in Figure 3, the information of GLSZM for EQD_2_ distribution is more abundant, which is a benefit for revealing the hidden correlation with RP incidence. Besides, the radio + eqdose model uses the first order, GLCM, and GLSZM features. The accumulated doses, the local dose variation, and the comprehensive information of direction, adjacent interval, and variation amplitude of dose distribution are used to construct the prediction model, so the evaluation metrics of radio + eqdose are better than the other three models in the study.

Previous studies demonstrated that the radio + dose model performed better than the DVH parameters for RP prediction [11–13]. In our study, the radio + dose model still performed well, and the radio + eqdose model was further investigated.

Fraction dose has been shown to be an essential factor in RP [29]. The impact of fraction dose can be illustrated using the well-known L-Q model. In this study, the fraction dose of the patients was from 1.5 to 2.75 Gy. It is defined as large-fractionated radiotherapy when the fractional dose exceeds 2.5 Gy. Compared with conventional fractionation radiation therapy, large-fractionated radiotherapy can increase the biological dose to improve tumor control and survival [42, 43]. The fraction dose of hyper-fractionated radiotherapy is generally lower than 2 Gy, more than once a day, and the interval time of irradiation is greater than 4 to 6 hours. The hyper-fractionated radiotherapy improves the local control rate of the tumor by increasing the total irradiation dose without significantly increasing the late reaction of normal tissue [44, 45]. When the total dose was 50 Gy, the EQD_2_ and BED were 54 Gy and 78 Gy for the 2.5 Gy fraction dose, 50 Gy and 72 Gy for the 2 Gy fraction dose, and 46 Gy and 66 Gy for the 1.5 Gy fraction dose. The differences in radio biology were significant.

The EQD_2_ model was used to solve the problem of equivalent dose calculation for different fractionated radiotherapy. Compared with the DVH, radio + DVH, and radio + dose model, the value of the training AUC, accuracy, and F1-score of radio + eqdose was higher, and the difference was statistically significant (*p* < 0.05). Besides, the average value of the recall and precision of radio + eqdose under 11 classifiers was higher than the other three models. The F1-score is the harmonic mean of precision and recall, reflecting the model's robustness. The results demonstrated that the radio + eqdose model could effectively improve the prediction ability of RP. The difference in testing AUC value was less than 1%, and the radio + eqdose model showed no obvious advantage. The possible reason is that the testing data are relatively small, so future studies will include more data to make the performance evaluation more objective. The single dose characteristics only utilize partial information on the dose distribution. Still, the multiomics method uses the info of CT image and dose distribution regarding the spatial relationship of voxels to train the prediction model. The advantages of the multi-omics method are apparent. Figure 3 showed that the information of GLSZM for EQD_2_ was more abundant than the physical dose, and we could extract more information from the EQD_2_ distribution for predicting RP.

The dose (absolute and relative dose) of the organ at risk can be obtained from DVH. However, the expression here is only the physical dose. Since the absorbed dose of the organ at risk is generally not 100%, the single dose is different from the conventional 2 Gy dose so that the EQD_2_ formula can calculate the isoeffect dose of the organ at risk. Because the tolerance of each organ at risk is obtained under conventional irradiation, it should be converted to EQD_2_ [32, 33]. Therefore, integrating the eqd-dosiomic features into training the model is reasonable. The experimental results also confirm the effectiveness of this method.

There are two main limitations of this study that are worth discussing. One is that the number of patients meeting the research requirements is relatively less. In order to avoid some errors due to equipment model, scanning conditions, and treatment planning, this study has only used the patients in our hospital. Besides, there are relatively few cases of RP ≥ 2 after radiotherapy in our hospital (15 patients). Therefore, the endpoint in this study was RP grade ≥ 1, which might limit its usefulness in actual clinical practice. In the next step, enrolling more patients in our hospital and multiple centers is necessary to validate our proposed approach. The other is that only radiomic and dosiomic features were considered in this study. However, RP is highly correlated with some clinical features, such as smoking history, disease stage, and tumor location. In future work, the clinical features will be added for further discussion. Finally, to prevent RP incidence, how to use the radiomic, dosiomic, and clinical characteristics to guide the design of radiotherapy treatment planning is the goal of our study.

## 5. Conclusion

In this study, we demonstrated that the multiomics model could improve the predictive performance of RP grade ≥ 1 compared to the DVH model. We also confirmed that the eqd-dosiomic features from the isoeffect dose distribution could improve the predictive model's performance with different fractionated radiotherapies. The multiomics method of radiomics and eqd-dosiomics could improve the predictive performance of RP. It is expected that further studies can be used to guide the design of radiotherapy treatment planning to realize individualized early intervention and treatment.

## Figures and Tables

**Table 1:** Patient clinical and treatment characteristics.

Clinical and treatment characteristics	Median (range)/*n* (%)
Age	62.5 (31–85)

*Sex*	
Male	83 (91%)
Female	8 (9%)

*Smoking history*	
No	37 (40%)
Smoking < 30 years	8 (9%)
Smoking ≥ 30 years	46 (51%)

*Stage*	
1	0
2	3 (3%)
3	63 (69%)
4	25 (28%)
Prescription dose	60 (40–66) Gy
Prescription dose per fraction	2 (1.5–2.75) Gy

*RP grade*	
0	52 (57%)
1	24 (26%)
2	9 (10%)
3	6 (7%)
4	0

**Table 2:** The evaluation indicators of four ML models under 11 classifiers (mean ± standard deviation).

Models	Training AUC	Testing AUC	Accuracy	Precision	Recall	F1
DVH	0.804 ± 0.053	0.801 ± 0.06	0.751 ± 0.068	0.738 ± 0.067	0.783 ± 0.16	0.751 ± 0.095
Radio + DVH	0.856 ± 0.044	0.805 ± 0.046	0.809 ± 0.062	0.772 ± 0.066	0.886 ± 0.118	0.821 ± 0.067
Radio + dose	0.833 ± 0.041	0.801 ± 0.057	0.806 ± 0.048	0.756 ± 0.045	0.911 ± 0.128	0.821 ± 0.061
Radio + eqdose	0.900 ± 0.033	0.799 ± 0.050	0.856 ± 0.034	0.809 ± 0.070	0.950 ± 0.059	0.870 ± 0.024

## Data Availability

The original contributions presented in the study are included in the article/Supplementary Material. Further inquiries can be directed to the corresponding authors.

## Supplementary Materials

Supplementary Materials Supplementary File 1: the clinical and treatment characteristics of 91 patients. File 2: the hyper-parameters for the eleven classifiers. File 3: the evaluation indicators of four ML models under 11 classifiers.
