# Supplementary material for: Machine Learning-Based Multiomics Prediction Model for Radiation Pneumonitis
Source: J Oncol. 2023 Feb 18;2023:5328927. doi: 10.1155/2023/5328927 (PMC9966572; doi:10.1155/2023/5328927)
Supplement: Supplementary Materials — Supplementary File 1: the clinical and treatment characteristics of 91 patients. File 2: the hyper-parameters for the eleven classifiers. File 3: the evaluation indicators of four ML models under 11 classifiers. [file 5328927.f1.zip › File 1_the clinical and treatment characteristics of 91 patients.pdf]

File 1_the clinical and treatment characteristics of 91 patients

patient	label	total dose (Gy)	fraction	fraction dose (Gy)	age	Sex (male 1; female 2)	smoking or not(yes 1; no 0)	smoking history (years)	TNM	RP grade
1	0	60	27	2.22	54	1	1	30	4	0
2	1	60	30	2	85	1	1	50	3	2
3	1	60	30	2	66	1	0	0	3	1
4	1	60	30	2	55	1	1	30	3	1
5	1	40	20	2	63	1	1	40	3	2
6	1	50	25	2	54	1	0	0	4	2
7	1	66	30	2.2	52	1	0	0	3	1
8	1	60	30	2	66	1	1	15	3	1
9	0	66	30	2.2	60	1	1	40	3	0
10	1	60	30	2	57	1	1	30	3	1
11	1	60	30	2	52	1	1	30	3	3
12	1	60	30	2	68	1	1	30	3	3
13	0	60	30	2	73	1	1	50	3	0
14	1	66	30	2.2	66	1	1	40	3	1
15	0	60	30	2	67	1	1	30	3	0
16	0	66	33	2	75	1	1	50	3	0
17	0	60	30	2	57	1	1	40	3	0
18	1	60	30	2	60	1	1	40	4	2
19	1	63	30	2.1	73	1	0	0	3	3
20	0	65	29	2.24	45	1	0	0	3	0
21	1	60	30	2	37	2	0	0	3	1
22	1	60	30	2	70	1	1	30	4	2
23	1	63	30	2.1	48	1	0	0	3	3
24	1	60	30	2	67	1	0	0	3	1
25	0	60	30	2	55	1	0	0	3	0
26	0	60	30	2	60	1	1	40	3	0
27	1	66	30	2.2	58	1	1	50	3	1
28	1	60	30	2	75	1	1	30	3	2
29	1	60	30	2	67	1	1	40	3	1
30	1	50	25	2	48	2	0	0	3	1
31	1	60	30	2	73	1	1	50	3	1
32	0	60	30	2	55	2	1	40	3	0
33	0	54	27	2	71	1	0	0	3	0
34	1	66	30	2.2	66	1	0	0	3	1
35	0	60	30	2	48	1	1	30	3	0
36	1	66	30	2.2	51	1	0	0	3	1

37	1	60	30	2	63	1	0	0	3	1
38	0	60	30	2	64	1	1	50	4	0
39	1	50	25	2	67	1	1	50	4	1
40	1	60	30	2	73	1	0	0	4	2
41	1	60	30	2	62	1	1	40	4	1
42	1	50	20	2.5	66	2	0	0	3	2
43	1	60	30	2	74	1	1	50	4	1
44	1	66	30	2.2	31	1	0	0	3	1
45	1	60	30	2	62	1	0	0	4	1
46	1	64	32	2	51	1	1	20	3	1
47	0	60	30	2	73	1	1	50	3	0
48	0	60	30	2	54	1	0	0	4	0
49	0	60	30	2	47	1	0	0	3	0
50	0	50	25	2	57	1	0	0	3	0
51	1	50	25	2	63	1	1	20	2	1
52	1	60	30	2	53	1	1	30	3	1
53	0	56	28	2	45	1	0	0	3	0
54	1	60	30	2	75	1	0	0	4	3
55	0	60	30	2	54	1	1	20	3	0
56	0	64	30	2.13	75	1	1	50	3	0
57	1	60	30	2	67	1	1	40	3	1
58	1	60	30	2	63	1	1	40	3	2
59	1	66	28	2.35	69	1	1	30	3	3
60	0	60	30	2	60	1	0	0	3	0
61	0	50	25	2	59	1	1	30	3	0
62	0	66	33	2	72	1	1	40	3	0
63	0	60	30	2	69	1	1	20	2	0
64	0	53	25	2.12	60	1	0	0	4	0
65	0	60	30	2	70	1	0	0	3	0
66	0	60	30	2	75	1	1	20	2	0
67	0	55	20	2.75	67	2	0	0	4	0
68	0	51	25	2.04	51	2	0	0	3	0
69	0	57	26	2.19	73	1	0	0	3	0
70	0	60	30	2	74	2	0	0	3	0
71	0	60	30	2	65	1	1	40	4	0
72	0	66	30	2.2	66	1	0	0	3	0
73	0	60	30	2	71	1	1	30	4	0
74	0	60	30	2	51	1	0	0	4	0
75	0	50	20	2.5	42	1	1	20	4	0
76	0	66	30	2.2	70	2	0	0	4	0
77	0	60	30	2	72	1	1	50	3	0
78	0	55	25	2.2	53	1	1	30	4	0
79	0	45	30	1.5	62	1	1	45	4	0

80	0	60	30	2	67	1	0	0	3	0
81	0	60	28	2.14	71	1	1	40	4	0
82	0	60	30	2	57	1	1	30	3	0
83	0	66	30	2.2	58	1	0	0	4	0
84	0	55	25	2.2	77	1	1	60	3	0
85	0	50	25	2	71	1	1	40	3	0
86	0	60	30	2	62	1	0	0	3	0
87	0	66	30	2.2	70	1	0	0	3	0
88	0	66	30	2.2	48	1	1	18	4	0
89	0	50	25	2	56	1	1	40	3	0
90	0	66	30	2.2	65	1	1	30	3	0
91	0	45	30	1.5	56	1	1	30	4	0
median		60	30	2	62.5					
max		66	33	2.75	85					
min		40	20	1.5	31					
