# Supplementary material for: Machine Learning-Based Multiomics Prediction Model for Radiation Pneumonitis
Source: J Oncol. 2023 Feb 18;2023:5328927. doi: 10.1155/2023/5328927 (PMC9966572; doi:10.1155/2023/5328927)
Supplement: Supplementary Materials — Supplementary File 1: the clinical and treatment characteristics of 91 patients. File 2: the hyper-parameters for the eleven classifiers. File 3: the evaluation indicators of four ML models under 11 classifiers. [file 5328927.f1.zip › File 2_the hyper-parameters for the eleven classifiers.pdf]

The hyper-parameters for the eleven classifiers

```
import os
import sys
import numpy as np
import pandas as pd
import matplotlib.pyplot as plt
from sklearn.model_selection import StratifiedKFold, cross_val_score, GridSearchCV

from sklearn.metrics import auc,confusion_matrix,plot_roc_curve

from sklearn.linear_model
import LogisticRegression,RidgeClassifier,Perceptron,PassiveAggressiveClassifier
from sklearn.ensemble import AdaBoostClassifier, RandomForestClassifier
from sklearn.neighbors import KNeighborsClassifier
from sklearn.naive_bayes import GaussianNB,MultinomialNB
from sklearn.svm import SVC
from sklearn.tree import DecisionTreeClassifier


model_params = {
    "LogisticRegression":{
        "model":LogisticRegression(),
        "params":{
                'penalty':['l1', 'l2', 'elasticnet', 'none'],
                'C':10.0**np.arange(-2,2),
                'solver' : ['newton-cg', 'lbfgs', 'liblinear', 'sag', 'saga'],
            }
    },
    "Ridge":{
        "model":RidgeClassifier(),
        "params":{
                'alpha':np.logspace(-3,2,10),
            }
    },
    "SVM":{
        "model":SVC(gamma='auto'),
        "params":{
                'kernel':['linear', 'poly', 'rbf', 'sigmoid'],
                'C':10.0**np.arange(-2,2),
            }
    },
```

```
"Perceptron":{
      "model":Perceptron(),
      "params":{
                  'penalty' : ['l2','l1','elasticnet'],
            }
},
"DecisionTree":{
      "model":DecisionTreeClassifier(),
      "params":{
                  'criterion':["gini", "entropy"],
            }
},
"RandomForest":{
      "model":RandomForestClassifier(n_estimators=100, n_jobs=-1),
      "params":{
                  'n_estimators':[10,50,100,200],
                  'criterion':["gini", "entropy"],
                  'oob_score' : [True,False]
            }
},
 "KNeighbors":{
      "model":KNeighborsClassifier(),
      "params":{
                  'weights' : ['uniform', 'distance'],
                  'n_neighbors':np.arange(3,8,1),
            }
 },
 "PassiveAggressive":{
      "model":PassiveAggressiveClassifier(),
      "params":{
                  'C':10.0**np.arange(-2,2),
                  'class_weight':["balanced",None],
            }
 },
 "GaussianNB":{
      "model":GaussianNB(),
      "params":{}
 },
 "MultinomialNB":{
      "model":MultinomialNB(),
      "params":{}
 },
 "AdaBoost":{
      "model":AdaBoostClassifier(),
```

```
                "params":{

'base_estimator':[DecisionTreeClassifier(max_features=None,class_weight="balanced
",max_depth=None)],
                    'n_estimators':[10,25,50,100],
                    'algorithm' : ['SAMME', 'SAMME.R'],
                    'learning_rate': np.arange(0.1,1,0.1)
                }
        },
}
```
