# Supplementary material for: Machine Learning-Based Multiomics Prediction Model for Radiation Pneumonitis
Source: J Oncol. 2023 Feb 18;2023:5328927. doi: 10.1155/2023/5328927 (PMC9966572; doi:10.1155/2023/5328927)
Supplement: Supplementary Materials — Supplementary File 1: the clinical and treatment characteristics of 91 patients. File 2: the hyper-parameters for the eleven classifiers. File 3: the evaluation indicators of four ML models under 11 classifiers. [file 5328927.f1.zip › File 3_the evaluation indicators of four machine learning models under 11 classifiers .pdf]

File 3_the evaluation indicators of four machine learning models under 11 classifiers

Model	Classifier	Training AUC	Testing AUC	Accuracy	Precision	Recall	F1
**DVH**	Logistic Regression	0.818265993	0.824175824	0.806818182	0.736842105	0.954545455	0.831683168
**DVH**	Ridge	0.818771044	0.879120879	0.795454545	0.75	0.886363636	0.8125
**DVH**	SVM	0.875224467	0.793956044	0.829545455	0.891891892	0.75	0.814814815
**DVH**	Perceptron	0.765824916	0.846153846	0.590909091	0.633333333	0.431818182	0.513513514
**DVH**	Decision Tree	0.729543471	0.703296703	0.727272727	0.7	0.795454545	0.744680851
**DVH**	Random Forest	0.872334456	0.870879121	0.784090909	0.755102041	0.840909091	0.795698925
**DVH**	KNeighbors	0.837289279	0.796703297	0.784090909	0.745098039	0.863636364	0.8
**DVH**	Passive Aggressive	0.777188552	0.835164835	0.704545455	0.8	0.545454545	0.648648649
**DVH**	GaussianNB	0.820173962	0.752747253	0.738636364	0.698113208	0.840909091	0.762886598
**DVH**	Multi NomialNB	0.827104377	0.807692308	0.795454545	0.732142857	0.931818182	0.82
**DVH**	AdaBoost	0.708331349	0.703296703	0.704545455	0.68	0.772727273	0.723404255
**Radio +DVH**	Logistic Regression	0.87037037	0.835164835	0.840909091	0.75862069	1	0.862745098
**Radio +DVH**	Ridge	0.852244669	0.835164835	0.806818182	0.728813559	0.977272727	0.834951456
**Radio +DVH**	SVM	0.898484848	0.796703297	0.852272727	0.860465116	0.840909091	0.850574713

**Radio +DVH**	Perceptron	0.776767677	0.840659341	0.659090909	0.675	0.613636364	0.642857143
**Radio +DVH**	Decision Tree	0.850485636	0.706043956	0.852272727	0.860465116	0.840909091	0.850574713
**Radio +DVH**	Random Forest	0.940993266	0.782967033	0.875	0.823529412	0.954545455	0.884210526
**Radio +DVH**	KNeighbors	0.833473342	0.796703297	0.784090909	0.745098039	0.863636364	0.8
**Radio +DVH**	Passive Aggressive	0.809736251	0.851648352	0.75	0.729166667	0.795454545	0.760869565
**Radio +DVH**	GaussianNB	0.890516274	0.824175824	0.818181818	0.733333333	1	0.846153846
**Radio +DVH**	Multi nomialNB	0.832042649	0.846153846	0.806818182	0.721311475	1	0.838095238
**Radio +DVH**	AdaBoost	0.861484514	0.741758242	0.863636364	0.863636364	0.863636364	0.863636364
**Radio +dose**	Logistic Regression	0.857547699	0.813186813	0.840909091	0.8125	0.886363636	0.847826087
**Radio +dose**	Ridge	0.792929293	0.824175824	0.818181818	0.74137931	0.977272727	0.843137255
**Radio +dose**	SVM	0.848148148	0.82967033	0.806818182	0.721311475	1	0.838095238
**Radio +dose**	Perceptron	0.801150393	0.840659341	0.670454545	0.702702703	0.590909091	0.641975309
**Radio +dose**	Decision Tree	0.806927743	0.706043956	0.806818182	0.8	0.818181818	0.808988764
**Radio +dose**	Random Forest	0.941105499	0.813186813	0.863636364	0.82	0.931818182	0.872340426
**Radio +dose**	KNeighbors	0.797079805	0.835164835	0.829545455	0.745762712	1	0.854368932
**Radio +dose**	Passive Aggressive	0.843602694	0.82967033	0.806818182	0.721311475	1	0.838095238
**Radio +dose**	GaussianNB	0.82815359	0.835164835	0.806818182	0.721311475	1	0.838095238

**Radio +dose**	Multi nomialNB	0.838664422	0.824175824	0.806818182	0.721311475	1	0.838095238
**Radio +dose**	AdaBoost	0.816660148	0.67032967	0.818181818	0.818181818	0.818181818	0.818181818
**Radio +eqddose**	Logistic Regression	0.947502806	0.747252747	0.897727273	0.906976744	0.886363636	0.896551724
**Radio +eqdose**	Ridge	0.899494949	0.708791209	0.852272727	0.781818182	0.977272727	0.868686869
**Radio +eqdose**	SVM	0.902132435	0.736263736	0.852272727	0.771929825	1	0.871287129
**Radio +eqdose**	Perceptron	0.886419753	0.796703297	0.852272727	0.771929825	1	0.871287129
**Radio +eqdose**	Decision Tree	0.905749292	0.813186813	0.909090909	0.928571429	0.886363636	0.906976744
**Radio +eqdose**	Random Forest	0.96087304	0.824175824	0.897727273	0.857142857	0.954545455	0.903225806
**Radio +eqdose**	KNeighbors	0.893873074	0.835164835	0.863636364	0.833333333	0.909090909	0.869565217
**Radio +eqdose**	Passive Aggressive	0.871997755	0.840659341	0.806818182	0.721311475	1	0.838095238
**Radio +eqdose**	GaussianNB	0.913945006	0.851648352	0.840909091	0.75862069	1	0.862745098
**Radio +eqdose**	Multi nomialNB	0.876318743	0.857142857	0.806818182	0.721311475	1	0.838095238
**Radio +eqdose**	AdaBoost	0.841662415	0.78021978	0.840909091	0.840909091	0.840909091	0.840909091
